# Subnitrate of Bismuth, in the Treatment of Burns and Scalds

**Published:** 1860-08

**Authors:** T. G. Richardson

**Affiliations:** One of the Attending Surgeons in the Charity Hospital, at New Orleans


### SUBNITRATE OF BISMUTH, IN THE TREAMENT OF BURNS AND SCALDS.

BY T. G. RICHARDSON, M. D., ONE OF THE ATTENDING SURGEONS IN THE CHARITY HOSPITAL, AT NEW ORLEANS.

To the thousand and one local remedies resorted to by medical men, as well as non-medical persons of both sexes, in the treatment of burns and scalds, I have the temerity to add another, if, indeed, I have not been anticipated by some one else. The substance referred to is the subnitrate of bismuth, which I employed during the past winter with remarkable success in the wards of the Charity Hospital. Heretofore I was in the habit of using the white-lead and linseed oil, as recommended first by Professor Gross, in the March number of Dr. John Bell's *Medical Bulletin* for 1844, and, although I consider it far superior to any other application in common use, I am now convinced by ample experience, that the bismuth is better. I was induced to give it a trial from a consideration of its well-known effect in calming irritation, and even actual inflammation, occurring in mucous membranes, the condition of these structures under such circumstances bearing a very close analogy to that of the skin after a burn of the first or second degree.

When I first began its use, I combined it with linseed oil in such proportions as to form a consistent paint, but subsequently substituted glycerin for the oil, and I am now inclined to think that the combination can never be surpassed, since by it every local indication is fully met. To prepare it, it is only necessary to rub the bismuth in a mortar with a sufficient amount of glycerin to form a paste or thick paint, which should be applied to the affected surface by means of a camel's-hair pencil, or a mop made of soft linen. Previously, however, to making the application, the parts should, if possible, be thoroughly dried, and for this purpose it is necessary to prick with a needle or other fine instrument, any blisters that may exist, and carefully wipe the surface, by gently pressing upon it a piece of dry lint or charpie. A thick coating having then been put on, the parts should be protected from the pressure or friction of the bed-clothes by covering it with a sheet of clean carded cotton or a layer of cotton batting, which may be confined, if necessary, by a thin bandage lightly applied. In burns of the first degree, one such application will often suffice, but in those of the second degree, it may be necessary to repeat it, in part at least, from day to day, in consequence of its disturbance and the wetting of the cotton by the subjacent discharges. The great object is to keep the denuded and tender surface entirely protected from atmospheric contact, which is especially important in the wards of hospitals where the air is always more or less loaded with noxious gases.

In slight burns of the first degree, in which there is only an erythematous inflammation without any vesication, the glycerin may be conveniently dispensed with and the dry bismuth dusted thick over the surface, in which case the watery exudations from the skin will form with it a pasty coating sufficiently protective. But still, the glycerin, possessing of itself properties powerfully soothing in their influence, should always be combined with it, unless there be some reasonable objection to its use.

The carded cotton or cotton batting I look upon as a most valuable adjuvant, and is superior to anything with which I am acquainted for warding off pressure.*—*North American Medico-Chirurgical Journal.*

* Since writing the above, Dr. W. Nichols, the highly accomplished House Surgeon of the Charity Hospital, has informed me that he has given the bis muth and glycerin a fair trial in a number of cases of burns under his charge, and freely accords to it all the praise which my experience has elicited in its behalf.

